# Incidental prostate ^18^F-FDG uptake without calcification indicates the possibility of prostate cancer

**DOI:** 10.3892/or.2014.3011

**Published:** 2014-02-04

**Authors:** HIROKO SEINO, SHUICHI ONO, HIROYUKI MIURA, SATOKO MOROHASHI, YUNYAN WU, FUMIYASU TSUSHIMA, YOSHIHIRO TAKAI, HIROSHI KIJIMA

**Affiliations:** 1 Department of Radiology and Radiation Oncology, Hirosaki University Graduate School of Medicine, Hirosaki 036-8562, Japan; 2 Department of Pathology and Bioscience, Hirosaki University Graduate School of Medicine, Hirosaki 036-8562, Japan

**Keywords:** ^18^F-FDG PET/CT, prostate cancer, prostate calcification, incidental prostate FDG uptake

## Abstract

Incidental ^18^F-fluorodeoxyglucose (^18^F-FDG) uptake in the prostate is often experienced in clinical practice; however, it is difficult to determine whether incidental uptake is indicative of a malignancy or benign state based on the maximum standardized uptake value (SUVmax). In the present study, we investigated the clinical significance of incidental prostate uptake by ^18^F-FDG positron emission tomography (PET)/CT, and examined the differences between malignant and benign uptake from a clinicopathological viewpoint. We reviewed 3,236 male subjects who underwent ^18^F-FDG PET/CT scans at Hirosaki University Hospital (Japan) from 2008 to 2012 in order to identify cases of incidental prostate FDG uptake. The final diagnosis was made by serum prostate-specific antigen (PSA) levels, biopsy, imaging studies and clinical follow-up with PET findings. Incidental FDG uptake of the prostate was observed in 53 cases (2%). Four cases were excluded due to insufficient clinical data, and 49 cases were included in the present study. Of the 49 cases, 8 (16%) had prostate cancer, while 41 (84%) were benign. All 8 malignant cases had high uptake areas, e.g. in the prostate peripheral zone, where there was no coexistence of calcification or FDG uptake. Of the 41 benign cases, 19 had high uptake in the inner zone, 17 in the peripheral zone, and 5 in both the inner and peripheral zones. Of the 41 cases, 18 (44%) showed FDG uptake coexisting with prostatic calcification. Incidental prostate ^18^F-FDG uptake infrequently signifies prostate cancer; however, FDG uptake not coexisting with calcification indicates the possibility of prostate cancer and should be included in the differential diagnosis for performing other clinical examinations.

## Introduction

Positron emission tomography (PET) is a method for determining biochemical and physiological processes by using radiopharmaceuticals labeled with positron-emitting radionuclides. FDG-PET has been widely applied to assess brain function, heart muscle metabolism and for the diagnosis of various tumors (1,2); however, the positive predictive value of prostate cancer has been considered to be low. A previous study reported that the maximum standardized uptake value (SUVmax) is not essential for differential diagnostic criteria of prostatic lesions (3). Prostate cancer shows no or mild FDG uptake because of its low glucose metabolism (4,6). In addition, FDG-PET is able to detect some prostate cancers due to urinary excretion of the radiotracer (5,6). FDG uptake is known to be unspecific to the cancer and may result from an inflammatory condition such as prostatitis (7). A previous study reported that FDG-PET shows 4.0% of histopathology-confirmed prostate cancer (8). On the other hand, cancer surveillance has revealed that the incidence of second primary cancers is 1.53–8.5% in patients with known first malignancies, and this incidence has risen due to the increase in the number of elderly patients and the prolonged cancer survival rate (9,10). Detection of second primary cancers is important, since they have a significant influence on patient management, particularly early cancers that require radical treatment (11).

Incidental uptake in the prostate is often experienced in clinical practice, but it is difficult to determine whether the uptake indicates a malignancy or a benign state based on the SUV alone. Only a few reports have described incidental prostate uptake on FDG-PET/CT (3). In the present study, we investigated the frequency and clinical significance of incidental prostate uptake on FDG-PET/CT and examined the relationship between the location of FDG uptake and prostate calcification.

## Materials and methods

### Patients

From May 2008 to August 2012, 3,236 male patients underwent ^18^F-fluorodeoxyglucose (^18^F-FDG)-PET/CT scans for various types of cancers at our hospital. Of the 3,236 PET/CT scan cases, 53 cases demonstrated incidental FDG uptake in the prostate. Of the 53 PET/CT scans, we analyzed the 49 cases demonstrating incidental FDG uptake with sufficient follow-up in this retrospective study, while 4 cases were excluded from the study due to insufficient follow-up.

### ^18^F-FDG PET/CT

In preparation for PET/CT, all patients fasted for at least 4 h, while water intake was encouraged. Delivered ^18^F-FDG (FDG scan injectable, 185 MBq on assay date; Nihon Medi-Physics, Co., Ltd., Tokyo, Japan) was injected intravenously and scanning was initiated 60 min later. During the 60-min uptake period, the patients drank a sufficient amount of water. A PET/CT system (Discovery ST Elite 16; GE Healthcare, Buckinghamshire, UK) was used to acquire all data in 7–8 bed positions, with an acquisition time of 2.5–3.0 min per bed position. CT was performed first (30–80 mA, 120 kV, 3.75–3.27 mm slice thickness). CT data were used for attenuation correction of PET data, as well as for co-registration with attenuation-corrected PET images. Then, PET data were acquired immediately from the same body region. PET, CT and fused PET/CT images were available for review and were displayed in axial, coronal and sagittal planes on a viewer system.

### ^18^F-FDG PET analysis

**Figure 1:**
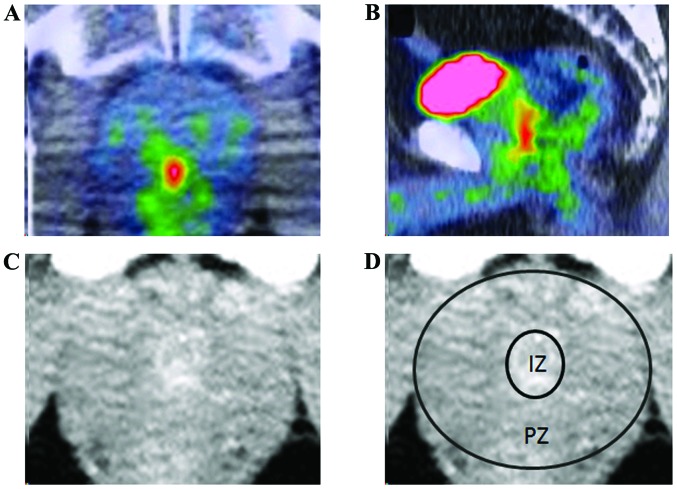
Image analysis. (A and B) Physiological uptake in the prostate urethra on coronal, sagittal and axial views was excluded. (C and D) To segment the inner zone and peripheral zone, we used the density difference in the CT portion of FDG-PET/CT. IZ, inner zone. PZ, peripheral zone.

FDG uptake in the prostate was visually defined as positive or negative. In the present study, physiological uptake in the prostate urethra on coronal, sagittal and axial views was excluded. The SUVmax for the prostate was obtained from transaxial views, and analyzed by the Mann-Whitney test (P<0.05, statistical significance). Prostate sites of FDG uptake (inner or peripheral zone) and patterns of FDG uptake (focal or diffuse) were evaluated on the axial view. The CT portion of the FDG-PET/CT was used to recognize the inner (prostate central gland: central + transition zones) and peripheral zones according to the contrast difference (Fig. 1).

### Prostate calcification analysis

**Figure 2:**
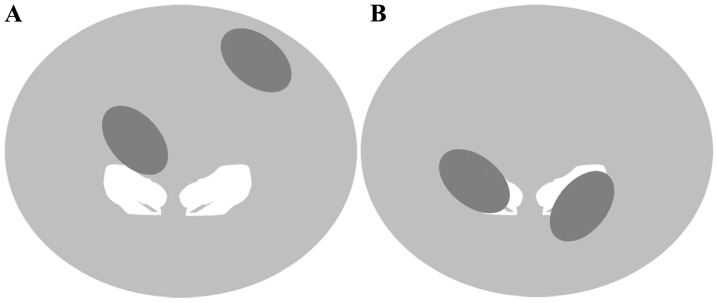
Prostate calcification analysis. The number and position of the prostate calcifications were not evaluated in the present study. (A) When prostate calcification and the FDG uptake area overlapped in less than 40% of the areas, the cases were defined as ‘uptake not coexisting with calcification’. (B) The other cases, i.e. 40% or more of overlapped areas, were defined as ‘uptake coexisting with calcification’.

When prostate calcification and the FDG uptake area overlapped in 40% or more of the areas, the cases were defined as ‘uptake coexisting with calcification’. The other cases, i.e. <40% of overlapped areas, were defined as ‘uptake not coexisting with calcification’ (Fig. 2). In the present study, the number and position of the prostate calcifications were not evaluated.

### Clinicopathological examinations

Final clinical diagnoses of the patients were determined based on the summarized results of the biopsy, serum prostate-specific antigen (PSA) levels, imaging studies (CT, MRI, follow-up PET/CT) and urological examinations. Urologists performed a biopsy for suspicious cases, and 12 patients underwent biopsy. In 28 cases, PSA levels were calculated after the PET/CT scan.

## Results

Of the 3,236 PET/CT images, incidental FDG uptake of the prostate was observed in 53 cases (2%), while 4 cases were excluded. These 49 cases were analyzed in the present study. Of the 49 cases, 8 (16%) had prostate cancer, and 41 (84%) were benign, i.e., the prostatic cancer discovery rate was 0.25% for all PET/CT scans. The SUVmax was not significantly different between the two groups.

### Malignant lesions

Of the 8 prostate cancer cases, 7 were ordinary adenocarcinomas and the other case was signet-ring cell carcinoma. All 7 cases of adenocarcinoma showed increased serum PSA levels, but the case of signet-ring cell carcinoma exhibited serum PSA levels within the normal range. The mean SUVmax of the 8 malignant lesions was 7.2±2.0. Prostate calcifications were present near the FDG uptake in 2 cases. All 8 cancer cases showed FDG uptake not coexisting with calcification, while the cancer lesions were located in the peripheral zone in 7 cases, and in both inner and peripheral zones in one case (Table I).

### Benign lesions

Of the 41 benign cases, 8 showed increased serum PSA levels, and 12 exhibited serum PSA levels within the normal range. PSA levels of the other 21 cases were not evaluated after PET/CT, but they were diagnosed as benign lesions by follow-up imaging studies or medical examination by urologists. The mean SUVmax of the 41 cases was 6.0±1.8. Eighteen cases (44%) exhibited FDG uptake coexisting with prostate calcifications, i.e. 13 of the 18 cases showed uptake in the inner zone and 5 in the peripheral zone. Of the 41 benign cases, 23 did not coexist with prostate calcification; there was FDG uptake in the inner zone in 6 cases, peripheral zone in 12 cases, and the inner to peripheral zone in 5 cases (Table II).

## Discussion

Our results demonstrated that abnormal hypermetabolic lesions in the prostate glands are not common (2%), and most were benign lesions (84%). The results are similar to those of previous reports (Table III); however, the incidence of prostate cancer in the present study was much different from previous studies. Cho *et al* (3) described incidental prostate uptake in 148 of 14,854 scans (1.0%), and prostate cancer in 9 of 67 subjects (13.4%) with further evaluations. Han *et al* (12) observed incidental prostate uptake in 63 of 5,119 scans (1.2%), and prostate cancer in 3 of 55 subjects (5.4%) with further evaluation. Hwang *et al* (13) observed incidental prostate uptake in 184 of 1,2037 scans (1.5%), and prostate cancer was found in 23 of 120 subjects (19.2%) with further evaluation. The different frequencies of incidental prostate cancer resulted from not only the large number of enrolled patients, but also the different confirmation methods. They also speculated that the incidence of prostate cancer detected by PET/CT might depend on the age and characteristics of the population, such as healthy people or patients with other cancers.

The incidence of prostatic cancer has recently increased in elderly people aged 60 and over. Tumor growth is gradual and it occurs frequently as multiple lesions. More than 95% of prostate cancer consists of adenocarcinoma histologically, and most prostate cancers arise from the peripheral zone (14). All of our carcinoma cases existed in the peripheral zone, whereas benign lesions existed in the inner zone.

**Figure 3:**
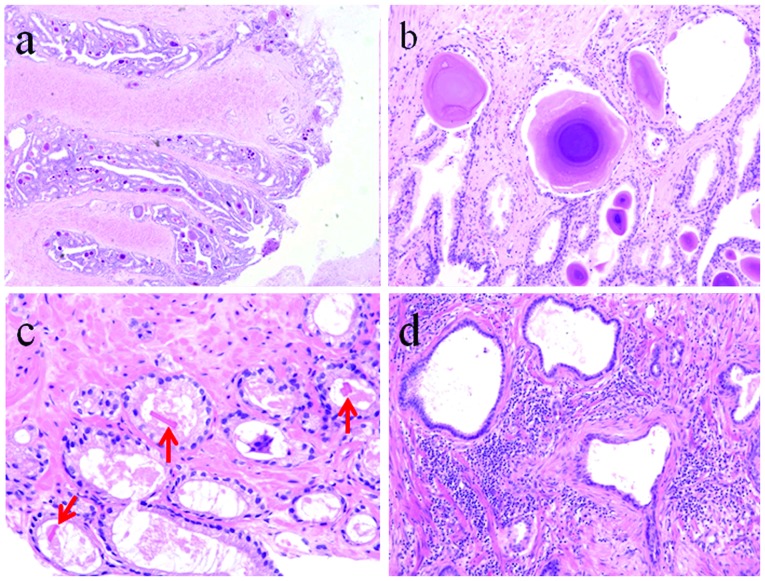
Histopathology of prostatic lesions. (a) H&E staining (magnification, ×12.5) shows multiple corpora amylacea at the prostatic urethra. (b) H&E staining (magnification, ×100) shows corpora amylacea of the layer structure. They are frequently observed in benign glands but are rarely noted in carcinomas. (c) H&E staining (magnification, ×100) shows prostatic crystalloids (arrows). They are often observed in malignant or atypical glands. (d) H&E staining (magnification, ×40) shows prostatitis with moderate lymphocyte infiltration.

**Figure 4:**
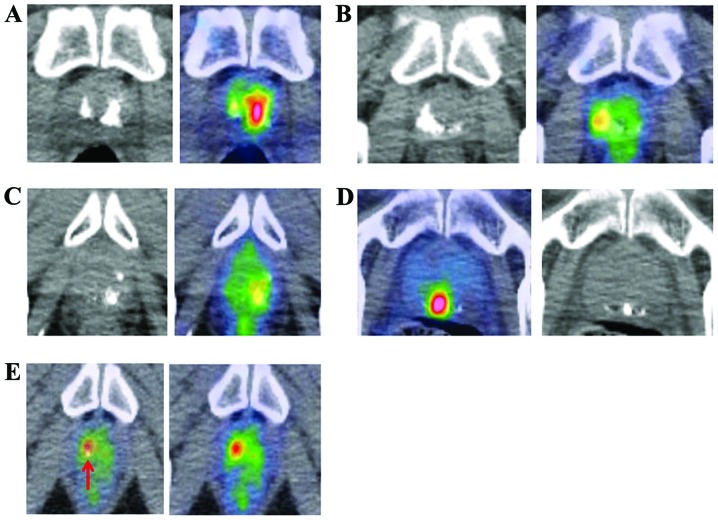
Benign uptake. All cases of FDG uptake coexisting with calcification were benign lesions irrespective of the site. (A–D) Calcification may be influenced by attenuation correction; therefore, changing window levels is needed for interpretation. Arrow shows calcification (E).

In elderly male patients, corpora amylacea, dense accumulations of calcified proteinaceous material, are frequently found in the prostatic ducts of elderly men (Fig. 3) and gradually form prostatic calcification, often without related clinical symptoms. Prostatic calcifications are associated with benign hyperplasia, chronic granulomatous prostatitis, and a history of chronic obstruction or stasis, such as in patients with urethral strictures and secondary intraprostatic reflux of urine (15–18). Bock *et al* (19) reported that prostatic calcifications were noted in 47.2% of men under 50 years of age and in 86% of men over 50 years of age. Histopathologically, corpora amylacea are frequently observed in benign glands but are rarely noted in carcinomas (20). On the other hand, prostatic crystalloid (intraluminal eosinophilic structures), intraluminal acidic mucin and intraluminal amorphous eosinophilic materials are less frequently found in both benign glands and adenocarcinomas (21–23). It is extremely important to determine corpora amylacea as criteria of benign lesions. Prostatic crystalloid (intraluminal eosinophilic structures), intraluminal acidic mucin, or intraluminal amorphous eosinophilic materials are invisible to CT because of their low density of calcification, while corpora amylacea are visualized by CT (20); therefore, FDG uptake coexisting with calcification visualized on CT is almost always consistent with benign findings (Fig. 4). On the other hand, FDG uptake without calcification in the peripheral zone is thought to possibly indicate a malignant lesion, but the differential diagnosis of benign/malignant prostatic lesions is not easy; and further clinical examinations such as serum PSA, MRI and biopsy are necessary to verify the diagnosis (24,25).

**Figure 5:**
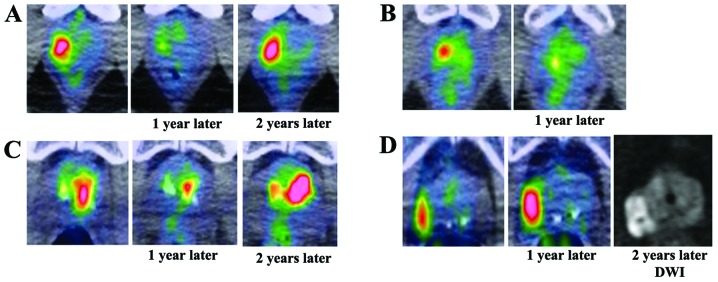
Reproducible FDG uptake. (A–C) All 3 cases were diagnosed as chronic inflammatory change such as prostatitis. Although SUVmax was different as assessed each year, the FDG uptake tended to exist in the same area. (D) One case was diagnosed as adenocarcinoma 2 years following the initial PET/CT scan. During those 2 years, biopsies were performed 3 times. DWI, diffusion weighted image.

FDG uptake is not only specific to cancer, but is also positive in inflammatory conditions such as prostatitis. Previous studies have reported that inflammation is associated with prostate cancer (26,27); however, reproducible FDG uptake in the same area is thought to be a potential malignant lesion, and careful observation is recommended (Fig. 5). In the present study, 4 patients showed reproducible FDG uptake in the same area and 1 patient had adenocarcinoma. In addition, distribution of the FDG uptake is important (15,28); FDG uptake in the inner area probably indicates benign uptake, since prostatic cancer frequently occurs in the peripheral zone, but not in the inner glands.

**Figure 6:**
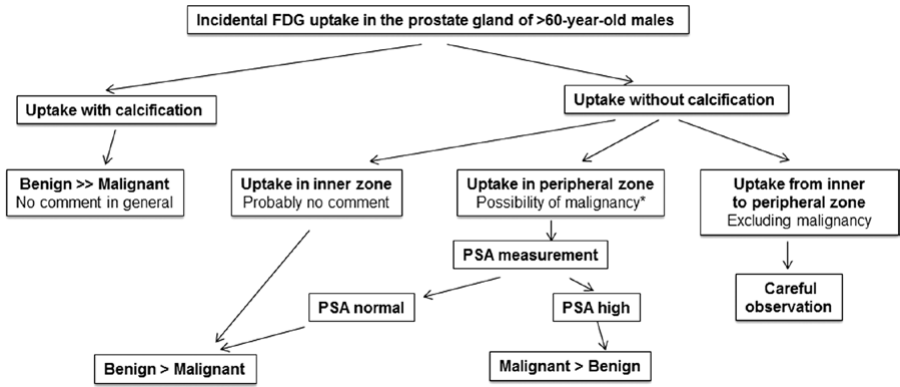
Diagnostic flow chart. Incidental FDG uptake in the prostate gland of males 60 years of age or older. *, Strong FDG uptake that occurs within a short period of time indicates probable inflammatory change.

Fig. 6 is a diagnostic flow chart of incidental FDG uptake in the prostate.

In conclusion, incidental FDG uptake in the prostate is an extremely rare finding in patients who undergo FDG-PET/CT. FDG uptake coexisting with calcification is indicative of a benign lesion; however, FDG uptake without calcification in the peripheral zone can indicate prostate cancer, and further examinations such as PSA, MRI and biopsy are necessary to exclude malignancy. Reproducible FDG uptake should be observed carefully since it can indicate malignancy or probable malignant potential.

## Figures and Tables

**Table I:** Malignant cases (n=8).

Patients	Age (years)	Indication for PET/CT	Gleason score	PSA (ng/ml)	SUVmax	Site	Calcification
Patient 1	57	Lung cancer	3+4	30.55	6.7	Peripheral	Absent
Patient 2	67	Bone tumor	4+5	85.20	9.0	Peripheral	Absent
Patient 3	73	Cholangiocarcinoma	4+4	8.23	5.7	Peripheral	Absent
Patient 4	76	Head and neck cancer	4+5	49.10	7.9	Peripheral	Present^a^
Patient 5	80	Head and neck cancer	4+5	31.00	7.3	Peripheral	Present^a^
Patient 6	61	Cancer screening	5+4	0.19	7.0	Peripheral	Absent
Patient 7	78	Lung cancer	4+5	19.71	3.7	Peripheral	Absent
Patient 8	66	Cancer screening	4+5	79.60	10.9	Peripheral-inner	Absent

a Prostatic calcification not coexisting with FDG uptake.

**Table II:** Benign cases: Uptake with or without calcification.

A, Uptake with calcification (n=18)			

Site	PSA unmeasured	PSA normal	PSA high
Inner zone (n=13)	10	2	1
Peripheral zone (n=5)	3	1	1

B, Uptake without calcification (n=23)			

Site	PSA unmeasured	PSA normal	PSA high

Inner zone (n=6)	4	1	1
Peripheral zone (n=12)	1	8	3
Both lobes (n=4)	3	0	1
Right lobes (n=1)	0	0	1

**Table III:** Summary of incidental FDG uptake in previously reported cases.

Authors/(ref.)	Subjects	Cases of incidental FDG uptake n (%)	Number of malignancies
Han *et al* (12)	5,119	63 (1.2)	3
Cho *et al* (3)	14,854	148 (1.0)	9
Hwang *et al* (13)	12,037	184 (1.5)	23
